# Effects of Hydrostatic Pressure on Electrical Retinal Activity in a Multielectrode Array-Based *ex vivo* Glaucoma Acute Model

**DOI:** 10.3389/fnins.2022.831392

**Published:** 2022-01-26

**Authors:** Claudia Ingensiep, Kim Schaffrath, Peter Walter, Sandra Johnen

**Affiliations:** Department of Ophthalmology, University Hospital RWTH Aachen, Aachen, Germany

**Keywords:** retina, RGC, pressure, glaucoma, MEA, taurine

## Abstract

Glaucoma is a heterogeneous eye disease causing atrophy of the optic nerve head (ONH). The optic nerve is formed by the axons of the retinal ganglion cells (RGCs) that transmit visual input to the brain. The progressive RGC loss during glaucoma leads to irreversible vision loss. An elevated intraocular pressure (IOP) is described as main risk factor in glaucoma. In this study, a multielectrode array (MEA)-based *ex vivo* glaucoma acute model was established and the effects of hydrostatic pressure (10, 30, 60, and 90 mmHg) on the functionality and survival of adult male and female wild-type mouse (C57BL/6) retinae were investigated. Spontaneous activity, response rate to electrical and light stimulation, and bursting behavior of RGCs was analyzed prior, during, and after pressure stress. No pressure related effects on spontaneous firing and on the response rate of the RGCs were observed. Even a high pressure level (90 mmHg for 2 h) did not disturb the RGC functionality. However, the cells’ bursting behavior significantly changed under 90 mmHg. The number of spikes in bursts doubled during pressure application and stayed on a high level after pressure stress. Addition of the amino sulfonic acid taurine (1 mM) showed a counteracting effect. OFF ganglion cells did not reveal an increase in bursts under pressure stress. Live/dead staining after pressure application showed no significant changes in RGC survival. The findings of our *ex vivo* model suggest that RGCs are tolerant toward high, short-time pressure stress.

## Introduction

Glaucoma is the second leading cause of blindness worldwide (Quigley, 1996; Quigley and Broman, 2006; Tham et al., 2014). It describes a heterogeneous disorder. Excavation (cupping) of the optic nerve head (ONH) and progressive loss of retinal ganglion cell (RGC) axons are characteristic for glaucoma disease. RGC loss causes corresponding visual field defects (Leske et al., 2003; Inatani et al., 2008) which in most cases is a painless process starting in the peripheral vision of the patient’s eye. Therefore, glaucoma often remains unnoticed in early stages. The pathogenesis is divided into mechanical (pressure related) and vascular (perfusion related) damage of the optic nerve. However, the main risk factor in glaucoma is an increased intraocular pressure (IOP). IOP mainly depends on the production and outflow of aqueous humor. Aqueous humor is secreted by the epithelium of the ciliary body into the posterior chamber. It subsequently enters the anterior chamber *via* the pupil by moving down an osmotic gradient. The trabecular meshwork forms the main outflow pathway (90%), next to the uveoscleral outflow (10%). Blockage of the outflow by ongoing humor production results in an increased IOP. Lowering the IOP by medical treatment in the form of eye drops, laser treatment or surgical treatment is considered the standard therapy.

Intraocular pressure in healthy human eyes is very individual, but usually ranges between 10 and 21 mmHg (Leske et al., 1997). Glaucoma patients often suffer from chronically increased IOP > 21 mmHg. During a glaucomatous attack, the IOP can even raise to > 60 mmHg. A glaucomatous attack is one of the most severe emergencies in ophthalmology. Patients suffer from a strong headache, nausea, and reduced and blurred vision in combination with a red eye. Nevertheless, increased IOP is not an obligatory characteristic of glaucoma. Some patients with normal tension glaucoma suffer from ONH atrophy at normal eye pressure, whereas other patients show an increased IOP without morphological damage.

Next to its mechanical load on the tissue, elevated IOP especially disturbs the blood flow in the eye (Howard and Sawyer, 1975). Direct compression of retinal blood vessels due to high IOP causes reduced blood flow when the ocular perfusion pressure is beyond its autoregulation capacity. The vascular stress results in a lack of oxygen (hypoxia). Due to their high metabolic activity, RGCs react sensitively to hypoxic stress. Therefore, hypoxic conditions lead to RGC activity loss and cell death (Winkler, 1981; Osborne et al., 2004).

To differentiate between the effects the stressors hypoxia and pressure have on the retina in glaucomatic disease, analyses under defined hypoxic conditions and defined pressure conditions are necessary.

The effect of hypoxic stress on the functionality and survival of RGCs has been demonstrated in our previous work (Ingensiep et al., 2021) as well as in multiple other studies (Gross et al., 1999; Kergoat et al., 2006; Janaky et al., 2007; Tan et al., 2011). RGCs transit into a stress mode, even after short-time hypoxia, where no spontaneous electrical activity and no response to electrical or light stimuli can be recorded. This effect is conditionally reversible; however, the survival rate of RGCs under hypoxic stress is very low.

In this study, the effect of pressure stress on RGCs was analyzed. We established a multielectrode array (MEA)-based *ex vivo* pressure model to analyze the retinal electrical activity prior, during, and after hydrostatic pressure application.

We also analyzed the effect of 2-aminoethanesulfonic acid (taurine) on RGC functionality and the survival rate of retinal cells during pressure stress. Taurine is a free amino sulfonic acid which is very abundant in mammalian tissue and especially highly concentrated in the retina (Macaione et al., 1974). It is involved in multiple physiological processes and proven to have neuromodulating and neuroprotective effects (Froger et al., 2012). Taurine is structurally similar to γ-aminobutyric acid (GABA) and has the ability to inhibit the excitatory effect of glutamate by binding to GABA receptors and therefore prevents excitotoxicity (Louzada et al., 2004). It is highly antioxidant, regulates osmotic pressure in cells, and affects the homeostasis of intracellular ion concentrations (El-Sherbeny et al., 2004). Taurine plays a crucial regulatory role in intracellular (cytoplasmic and intra-mitochondrial) calcium (Ca^2+^) transport by stimulating ATP-dependent Ca^2+^ uptake at low Ca^2+^ concentration [Ca^2+^] and lowering the uptake at high [Ca^2+^] (Pasantes-Morales and Ordonez, 1982; El Idrissi, 2008). This mechanism also affects Ca^2+^-dependent mitochondrial pores (mPTPs). Apoptosis-induced factors, such as cytochrome c, use these pores to get into the cytosol. Therefore, mitochondria-mediated apoptosis can be prevented by taurine.

## Materials and Methods

### Animals

Male and female C57BL/6J wild-type (wt) mice aged 12–20 weeks from Janvier (Le Genest-Saint-Isle, France) were kept under controlled light conditions (12:12 h light/dark cycle), at a room temperature of 21–23°C, and a humidity of 35–65% at the Institute of Laboratory Animal Science (Faculty of Medicine, RWTH Aachen University). Water and food were available *ad libitum* and cages were cleaned once a week. For sacrifice, mice were deeply anesthetized with isoflurane (AbbVie, Wiesbaden, Germany) and killed by decapitation. All experiments were performed after approval was obtained by the regulatory authorities and in accordance with the ARVO statement for the Use of Animals in Ophthalmic and Vision Research and the German Law for the Protection of Animals.

### Medium

Ames’ medium (Sigma-Aldrich, St. Louis, MO, United States) (Ames and Nesbett, 1981) was dissolved in water, bubbled with 100% CO_2_ for 30 min at room temperature (RT), and supplemented with sodium bicarbonate. The medium was adjusted to a pH of 7.4–7.5 with sodium hydroxide and continuously bubbled with carbogen gas (95% O_2_, 5% CO_2_). Taurine was dissolved in Ames’ medium to a concentration of 1 mM (Chen et al., 2009; Froger et al., 2012). NMDA receptor antagonist DL-2-Amino-5-phosphonopentanoic acid (DL-AP5, 50 μM) and AMPA/kainate receptor antagonist 6-cyano-7-nitroquinoxaline-2,3-dione (CNQX, 20 μM) were freshly prepared and added to the Ames’ medium in order to block RGC glutamate receptor input (Biswas et al., 2014).

### Retina Preparation

Retina preparation was performed as previously described (Ingensiep et al., 2021). Briefly, both eyes of an animal were enucleated directly after sacrifice and put into freshly carbogenated Ames’ medium. The left eye was pierced at the limbus with a 27 G cannula and cut radially half open. It was kept separately in freshly carbogenated Ames’ medium as backup for later use if the MEA experiment with the right eye failed. The right eye was opened at the limbus with an encircling cut. The anterior segment and lens were removed and the retina was carefully detached from the eye cup and separated by a cut through the optic nerve. The vitreous body was carefully removed completely using forceps. The retina was cut into a square shape and mounted on a nitrocellulose frame with RGCs facing up. The frame was placed onto the electrode field of the MEA with RGCs facing down.

### Multielectrode Array Setup

The MEA2100 system from Multi Channel Systems (Reutlingen, Germany) was used to perform electrophysiological recordings (chapter Multielectrode Array Recordings) of murine retinae. It comprised a head stage with an integrated preamplifier, which was used for recording as well as for stimulation, and an interface board that served as digital/analog converter transmitting data in real time. A MEA was placed in the head stage that was connected to the interface board, which was connected to a personal computer (PC). The setup was placed on an air-suspended table (Ametek, Berwyn, PA, United States) in a faraday cage (Ametek) to minimize noise caused by vibrations and electronic devices.

60MEA200/30iR-Ti-pr-T type MEAs (Multi Channel Systems) were used. The MEAs possess 60 titanium nitride (TiN) electrodes arranged in a square field of 8 × 8 electrodes with the four electrodes at the corners being spared out and one electrode serving as reference, resulting in 59 electrodes for recording and stimulation. The electrodes were 30 μm in diameter and positioned with a distance of 200 μm to each other. All MEAs had a plastic ring around the electrode field with an inner diameter of 26 mm and a thread on the inside. Before every experiment, MEAs were hydrophilized with oxygen plasma for 2 min at 0.5 mbar in a plasma cleaner (Diener Electronic, Ebhausen, Germany).

### Pressure Stress

Hydrostatic pressure was applied to the retina by adding a custom-made pressure lid to the MEA setup. The lid was designed and fabricated in the in-house scientific workshop (*Wissenschaftliche Werkstatt*, University Hospital RWTH Aachen). It was screwed onto the inner thread of the ring of the MEA, forming a leak-proof chamber. The lid was connected to a gravitation based VC^3^ perfusion system (ALA Scientific Instruments, Farmingdale, NY, United States) that provided the retina with freshly carbogenated medium during the experiments (inflow). The outflow was regulated by a flow control yielding a perfusion rate of 3 ml/min. The pressure inside the chamber was directly dependent on the height of the medium tube of the perfusion system. By raising the fluid column, the hydrostatic pressure inside the chamber increased. In order to keep the fluid column level during perfusion, an additional medium reservoir was installed on top of the medium tube, filling it with the same perfusion rate.

Access points in the lid for O_2_-, pH-, and pressure sensors enabled monitoring of the experimental conditions inside the chamber during MEA recordings. Oxygen concentration [O_2_] was measured by the OxyMicro sensor and recorded *via* OxyMicro software (World Precision Instruments, Sarasota, FL, United States). The pH was monitored by the pHOptica micro sensor and recorded *via* pHOptica software (World Precision Instruments). One measurement per minute was taken for [O_2_] and pH, respectively. Pressure was continuously measured by an Xtrans pressure transducer (Codan, Lensahn, Germany) and displayed by a connected IntelliVue MP30 Anesthesia patient monitor (Philips, Hamburg, Germany). The aim pressure ± 1 mmHg was tolerated.

**FIGURE 1:**
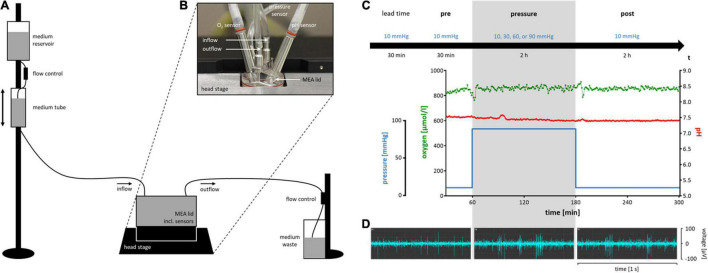
Schematic diagram of the experimental setup of the pressure model and timeline with representative experimental conditions and RGC activity. **(A)** A simplified sketch of the experimental setup for the pressure model (not to scale). **(B)** The MEA lid on top of a MEA. The five integrated access points for in-, and outflow of the perfusion system, and for pressure, O_2_, and pH sensors are shown. **(C)** The experimental timeline including a lead time of 30 min at 10 mmHg, the pre-phase of 30 min at 10 mmHg, the pressure-phase of 2 h at either 10 (control), 30, 60 or 90 mmHg, and the post-phase/recovery time of 2 h at 10 mmHg. Below the timeline, representative examples of pressure level, oxygen concentration, and pH of an experiment with 90 mmHg are shown. Note that during changes in the pressure level, [O_2_] and pH remain stable. **(D)** Representative examples of the electrical spontaneous RGC activity of a wt mouse retina in the pre-, pressure-, and post-phase of an experiment with 90 mmHg.

Figures 1A,B show a schematic diagram of the experimental setup and a picture of the pressure lid. Representative examples for pressure, [O_2_], and pH over time are shown in Figure 1C. Examples of the RGC firing behavior under different pressure levels are shown in Figure 1D. The spontaneous electrical activity of RGCs as well as their responses to given stimuli (electrical and light) persisted under pressure and could be recorded throughout the experiment (pre, pressure, post). The control pressure was set to 10 mmHg according to the normal eye pressure of mice (Kim et al., 2007; Lei et al., 2011). Pressure levels of 30, 60, and 90 mmHg were tested to simulate an increased intraocular pressure. 30 mmHg (Choi et al., 2015) were chosen as moderate pressure increase since a commonly investigated mild pressure level of 20 mmHg (Aihara et al., 2014; Pang et al., 2015) did not reveal any effects on the RGC firing behavior. 60 mmHg (Bui et al., 2005; Osborne et al., 2015) were chosen as high pressure increase that can occur during glaucomatous attacks. 90 mmHg (Aihara et al., 2014; Osborne et al., 2015) were investigated to emphasize the slight effects that were measured under 60 mmHg. In order to rule out an effect of the pressure lid itself, two additional controls were performed: a control without any pressure application (0 mmHg) and a control using the conventional MEA setup without any lid (open MEA).

Every experiment started with a lead time of 30 min under control conditions (10 mmHg) (Figure 1C) to allow the retina to recover from the preparation stress and let electrical activity reach a steady state. After the lead time, recordings were started. If spontaneous RGC firing was detected on less than ten of the overall 59 recording channels after lead time, the retina was rejected. After additional 30 min under control conditions (pre-phase), the test pressure was applied for 2 h (pressure-phase). A recovery time of 2 h at 10 mmHg completed the experimental timeline (post-phase).

### Multielectrode Array Recordings

The electrical activity of the RGCs was displayed and recorded with Multi Channel Experimenter software (Multi Channel Systems) at a sample rate of 25,000 Hz. Raw data was filtered with a second order Butterworth high pass filter with a cutoff at 200 Hz (filter 1) and a second order Butterworth low pass filter with a cutoff at 2,000 Hz (filter 2). A spike detector used the band pass filtered data from filter 1 and 2 to identify action potentials at a threshold of −20 μV. If necessary, noise channels had to be excluded from spike detection. Measurements of 60 s each were performed, recording either the spontaneous firing of the cells or their response to stimulation.

#### Electrical Stimulation

For each experiment, two different stimulation electrodes were chosen and used in sequence. During a stimulation recording, only one electrode stimulated the retina. A biphasic current pulse (± 80 μA, 500 μs per phase) with the cathodic phase preceding the anodic phase was used. Five pulses with an interstimulus interval (ISI) of 10 s were given per recording. Electrical stimulation was used to roughly investigate the RGC functionality. For a more refined analysis, light as the physiological stimulus of the retina was used.

#### Light Stimulation

For experiments with light stimulation, mice were dark adapted for 1 h before sacrifice. Preparation and the experiment itself were performed in the dark under dim red light. The LED stimulator MEA2100-opto-stim and Stimulus generator STG4002-1.6A-opto (Multi Channel Systems) were added to the MEA setup. A neutral white 4100K LED (Quadica Developments Inc., AB, Canada) was positioned underneath the MEA, illuminating the retina from the ganglion cell layer (GCL). The LED was programmed with Multi Channel Stimulus ll software and stimulation time points were detected and recorded with Multi Channel Experimenter. Full-field light pulses (1 s, 10 s ISI) were generated five times per recording. The LED was run with 500 mA, resulting in a luminous flux of 160 lm.

### Live/Dead Staining

To investigate the number of dead cells in different retinal layers after pressure stress, retinae were live/dead double stained with fluorescein diacetate (FDA) (Merck, Darmstadt, Germany) and propidium iodide (PI) (ICN Biomedicals, Costa Mesa, CA, United States). FDA enters the cellular membrane of living cells and is metabolized by esterases, resulting in green fluorescent somata. PI can only penetrate the disrupted membrane of dead cells and binds to DNA, resulting in red fluorescent nuclei. Three different groups, 10 mmHg (control), 90 mmHg, and 90 mmHg + taurine, were investigated. The pressure application was performed according to the setup and timeline of the MEA experiments excluding lead time and post pressure phase. However, some changes had to be done to ensure that the retinae could be removed from the pressure chamber after an experiment without causing any damage to the tissue. MEAs were not hydrophilized in advance and retinae were placed on the electrode field with the nitrocellulose frame facing down and RGCs facing up to prevent the tissue from sticking to the MEA. A metal ring with a nylon mesh was placed on top of the retinae, preventing the tissue from swimming freely inside the chamber. For staining experiments, both retinae of an animal were treated simultaneously. After pressure treatment, each retina was added to 600 μl of staining solution [20 μl FDA (5 mg/ml), 20 μl PI (1 mg/ml) in 1.2 ml Ames’ medium] in a 24-well plate (Eppendorf, Hamburg, Germany). The well plate was put into a metal box and placed onto an orbital shaker (Heidolph Instruments, Schwabach, Germany). The dyes were applied for 5 min at 60 rpm to ensure that the tissue was completely saturated by the solution. Subsequently, the tissue was washed three times for 5 min in freshly carbogenated Ames’ medium. To prevent bleaching and an increasing number of dead cells due to the acetone in the FDA solution, all steps were performed quickly, protected from light, and immediately before imaging. For positive PI controls, retinae suffered hypoxic stress for 4 h and were afterward stained using the same protocol.

### Two-Photon Laser Scanning Microscopy

The live/dead double stained retinae were analyzed *via* two-photon laser scanning microscopy (TPLSM) as described earlier (Ingensiep et al., 2021). Imaging was done with a two-photon microscope (Olympus Fluoview FV1000 MPE, Olympus Corporation, Tokyo, Japan) attached to a pulsed Ti:Sapphire laser (MaiTai DeepSee, SpectraPhysics, Santa Clara, CA, United States). A 25x NA 1.05 water dipping objective was used. Representative regions in the superior, inferior, nasal or temporal retina beyond the ONH and in between the main blood vessel branches were chosen and laser intensity was adjusted. Two stacks of subsequent images (xy-frames, 1,024 pixel × 1024 pixel) over depth (z, ∼150 μm) with a z-step of 1 μm were recorded for each retina.

### Data Analysis

#### Multielectrode Array Recordings

Since one MEA electrode could detect the electrical activity of not only one, but several cells, spike sorting was mandatory to investigate the firing behavior on single cell level. Offline Sorter software version 4 (Plexon Inc., Dallas, TX, United States) was used to perform spike sorting *via* a principal component analysis (PCA). Spikes were clustered according to their characteristic waveforms and marked as units with one unit representing one cell.

Data was further processed with NeuroExplorer software version 5 (Nex Technologies, Colorado Springs, CO, United States). Firing frequency, response rate, spike amplitude, and bursting behavior were analyzed and exported into Excel by operating NeuroExplorer with a custom-written Python script.

The response rate for electrical stimulation was analyzed for cells on the eight recording channels surrounding the stimulation electrode. It was defined as the coefficient of the number of spikes 3 s before the stimulus and 0.5 s after the stimulus. For full-field light stimulation, the response rate was calculated for cells on all recording channels. The spike rate around the start (onset) and stop (offset) of the light pulse was investigated. The number of spikes 1 s before and 0.5 s after stimulus onset and offset, respectively, were analyzed and the coefficient was calculated. According to their response rate, RGCs were roughly categorized into ON, ON-OFF, and OFF cells. If the response rate to the onset was ≥ 1.5 and the response rate to the offset < 1.5, the cell was categorized as ON. If the response rate to the onset as well as to the offset was ≥ 1.5, the cell was categorized as ON-OFF. If the response rate to the onset was < 1.5 and the response rate to the offset ≥ 1.5, the cell was categorized as OFF. If both response rates were < 1.5, the cell remained uncategorized.

A burst analysis identified spikes in bursts with interval algorithm parameters of a 0.01 s maximal interval to start a burst, a 0.03 s maximal interval to end a burst, a 0.02 s minimal interval between bursts, a 0.01 s minimal duration of a burst, and a minimal number of three spikes within a burst.

#### Live/Dead Staining

Image analysis was performed in Imaris software version 9 (Bitplane, Oxford Instruments plc., Abingdon, United Kingdom). The recorded image stacks were 3D rendered and divided into three regions of interest (ROIs): GCL, inner nuclear layer (INL), and outer nuclear layer (ONL). The number of dead cells (red channel) was automatically counted for every ROI using the *Spot* function of the software. Virtual sections of the image stacks in x, y, and z direction were made for each group (10 mmHg, 90 mmHg, 90 mmHg + taurine).

### Experimental Design and Statistical Analyses

For MEA recordings, a number of five animals per group was chosen. MEA experiments with addition of glutamate receptor antagonists were performed with three animals. Each group contained both male and female mice. 14 retinae of seven animals had to be rejected for not meeting the experimental criteria, resulting in an overall number of 50 mice (21 male, 29 female) used for MEA recordings.

Twelve retinae of three male and three female mice were used for the staining experiments, resulting in eight image stacks per group.

Design of graphs and statistical analyses were performed using GraphPad Prism version 7 (GraphPad Software Inc., San Diego, CA, United States). A *p*-value < 0.05 was considered significant. In figures, different significance levels are represented by asterisks: *p < 0.05, ^**^p < 0.01, ^***^p < 0.001, ^****^p < 0.0001. Outliers were identified *via* robust regression and outlier removal (ROUT) with a ROUT coefficient (Q) of *Q* = 0.1%. Mean values presented in graphs with identified outliers refer to the mean of the cleaned data. Mean values presented in the text include all data and are presented as mean ± standard deviation (SD).

## Results

### Multielectrode Array Recordings

**FIGURE 2:**
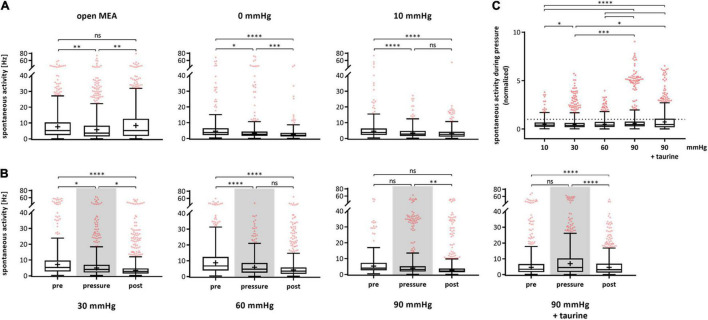
Spontaneous activity of RGCs under different pressure levels. The spontaneous firing frequency [Hz] of RGCs before (pre), during (pressure), and after (post) pressure stress is shown. **(A)** The results of the control experiments without pressure lid (open MEA), 0 mmHg, and 10 mmHg are presented. **(B)** The results of the test groups with 30, 60, 90 mmHg, and 90 mmHg + taurine (1 mM) are presented. **(C)** Summary of the normalized spontaneous RGC activity during pressure is shown. The activity before pressure (pre) was set to 1. Data are presented as box and whisker (min to max) plots. Outliers calculated by ROUT (*Q* = 0.1%) are shown as red circles. Mean values of the cleaned data are shown as + . Significant values are indicated by asterisks. Non-significant values are labeled with ns in panels **(A,B)** and not labeled in panel **(C)**. One-way ANOVA with *post hoc* Tukey’s test was performed with GraphPad Prism Software version 7. **(A)** *F*(2, 1447) = 6.965, *p* = 0.0010; *F*(2, 847) = 18.03, *p* < 0.0001; *F*(2, 939) = 24.55, *p* < 0.0001; **(B)** *F*(2, 1370) = 14.95, *p* < 0.0001; *F*(2, 1495) = 42.19, *p* < 0.0001; *F*(2, 1432) = 6.752, *p* = 0.0012; *F*(2, 1550) = 18, *p* < 0.0001; **(C)** *F*(4, 2467) = 20.54, *p* < 0.0001. *n*_retinae_ = 5/group, *n*_cells_ = 152–430/group, *n*_recordings_ = 16/experiment.

The spontaneous RGC firing frequency on single cell level after spike sorting was analyzed and plotted for each of the experimental phases pre, pressure, and post (Figure 2). For control experiments, the same temporal phases were investigated; however, no pressure change was applied (Figure 2A). In the control group without pressure lid (open MEA), the mean firing frequency decreased by 23% from 11.36 ± 12.08 Hz (pre) to 8.76 ± 10.78 Hz (pressure) and increased by 23% to 10.80 ± 11.78 Hz during the post-phase. In the 0 mmHg control group, the mean firing frequency of the RGCs decreased by 27% from 8.00 ± 11.13 Hz (pre) to 5.81 ± 10.03 Hz (pressure) and by 43% to 3.32 ± 4.66 Hz (post). In the control group with a pressure level of 10 mmHg, the mean firing frequency decreased by 43% from 7.13 ± 9.71 Hz (pre) to 4.08 ± 3.97 Hz (pressure) and by 8% to 3.75 ± 4.25 Hz (post). With an increased pressure of 30 mmHg, the mean firing frequency decreased by 21% from 10.90 ± 12.61 Hz to 8.59 ± 10.84 Hz and further decreased by 21% to 6.79 ± 9.74 Hz during the post-phase (Figure 2B). Under a pressure of 60 mmHg, the mean firing frequency decreased by 40% from 11.66 ± 11.39 Hz to 7.02 ± 6.75 Hz and further decreased by 9% to 6.42 ± 8.22 Hz. Under an increased pressure of 90 mmHg, the mean RGC firing frequency was not significantly different between the pre- (6.86 ± 7.56 Hz) and pressure-phase (7.21 ± 10.26 Hz), but decreased during the post-phase by 26% to 5.30 ± 8.44 Hz.

Spontaneous firing frequency was not affected by taurine. As in the corresponding MEA experiments with 90 mmHg without taurine, the spontaneous firing frequency decreased over time by 34% (pre: 9.56 ± 13.78 Hz, pressure: 9.56 ± 11.42 Hz, post: 6.34 ± 7.47 Hz). Figure 2C sums up the spontaneous RGC activity during pressure stress and compares the different pressure levels. The normalized firing frequency is shown with the frequency during the respective pre-phase being set to 1. The mean RGC firing frequency under 10 mmHg was 57% of the initial frequency. Under 30 mmHg, the frequency was 79%, under 60 mmHg 60%, and under 90 mmHg 105%. However, no pressure dependent effect on the spontaneous activity of the RGCs was found. With the addition of taurine, the frequency was 100% under 90 mmHg.

The unsorted MEA data, representing RGC activity on recording channel/electrode level, showed an increase in spontaneous firing under 90 mmHg pressure. The spontaneous activity increased by 82% from 9.68 ± 15.10 Hz (pre) to 17.58 ± 23.60 Hz (pressure) and by 11% to 19.43 ± 31.18 Hz (post) (data not shown).

**FIGURE 3:**
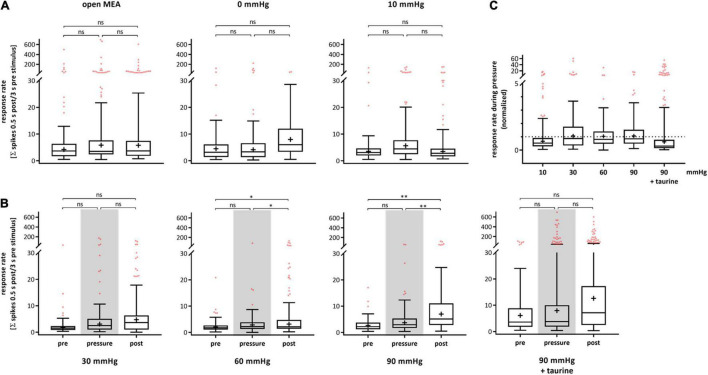
Response rate of RGCs after electrical stimulation under different pressure levels. The RGC response to electrical stimulation (± 80 μA, 500 μs per phase) before (pre), during (pressure), and after (post) pressure stress is shown. The response rate was defined by the coefficient of the number of spikes 0.5 s after stimulation and 3 s before stimulation. **(A)** The results of the control experiments without pressure lid (open MEA), 0 mmHg, and 10 mmHg are presented. **(B)** The results of the test groups with 30, 60, 90 mmHg, and 90 mmHg + taurine (1 mM) are presented. **(C)** Summary of the normalized RGC response rate during pressure is shown. The response rate before pressure (pre) was set to 1. Data are presented as box and whisker (min to max) plots. Outliers calculated by ROUT (*Q* = 0.1%) are shown as red circles. Mean values of the cleaned data are shown as + . Significant values are indicated by asterisks. Not significant values are labeled with ns in panels **(A,B)** and not labeled in panel **(C)**. One-way ANOVA with *post hoc* Tukey’s test was performed with GraphPad Prism Software version 7. **(A)** *F*(2, 387) = 0.0403, *p* = 0.9605; *F*(2, 160) = 0.4285, *p* = 0.6522; *F*(2, 187) = 0.5299, *p* = 0.5896; **(B)** *F*(2, 224) = 2.654, *p* = 0.0726; *F*(2, 278) = 4.854, *p* = 0.0085; *F*(2, 240) = 2.257, *p* = 0.1069; *F*(2, 482) = 2.155, *p* = 0.1170; **(C)** *F*(4, 531) = 2.206, *p* = 0.0671. *n*_retinae_ = 5/group, *n*_cells_ = 47–118/group, *n*_recordings_ = 19/experiment.

The RGC response to electrical stimulation (± 80 μA, 500 μs per phase) on single cell level after spike sorting was analyzed and plotted for each experimental phase (Figure 3). Figure 3A shows the response rates for the control experiments. The open MEA control revealed no significant changes in the RGC response rate (pre-phase: 19.00 ± 63.61, pressure-phase: 21.77 ± 80.06, post-phase: 20.79 ± 61.51). In the 0 mmHg control, the RGC response rate changed by 41% from 8.92 ± 19.04 to 12.59 ± 33.07 and by 26% to 9.26 ± 9.08. The 10 mmHg control revealed an increase by 56% from 8.44 ± 20.58 to 13.12 ± 24.00 and by 8% 12.10 ± 26.60. Figure 3B shows the results of the test groups. The mean RGC response rate in the 30 mmHg group increased by 228% from pre- (2.89 ± 4.53) to pressure- (9.49 ± 26.64) and by 2% to post-phase (9.71 ± 17.57). Under 60 mmHg, the response rate increased by 44% from 2.74 ± 3.08 to 3.94 ± 8.31 and further increased by 106% to 8.10 ± 17.30. Under 90 mmHg, the RGC response rate also increased by 50% from 3.47 ± 3.37 to 5.22 ± 8.01, and by 115% to 11.21 ± 19.03 after pressure stress. With addition of taurine, the response rate after electrical stimulation strongly increased by 237% during pressure and remained at a high level after pressure stress (pre: 12.77 ± 22.47, pressure: 43.12 ± 108.70, post: 42.43 ± 112.90).

Figure 3C shows a summary of the normalized response rate during pressure stress for each pressure level with the initial response rate before pressure stress being set to 1. The RGC response rate to electrical stimulation was 156% under 10 mmHg, 328% under 30 mmHg, 144% under 60 mmHg, 150% under 90 mmHg, and 337% under 90 mmHg with the addition of taurine. Overall, we observed big standard deviations throughout all groups and phases revealing a high variance of the RGC response to electrical stimulation in general. However, no significant pressure dependent effect on the RGC response was found.

**FIGURE 4:**
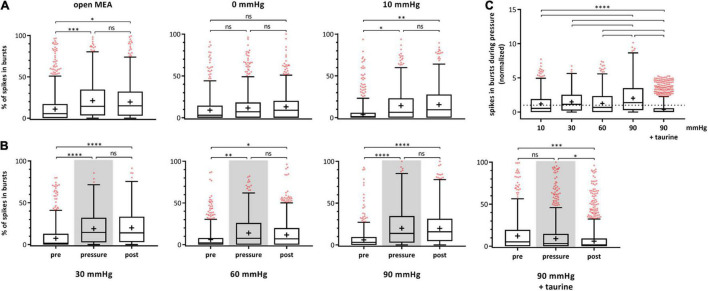
Bursting behavior of RGCs under different pressure levels. The number of RGC spikes in bursts [%] before (pre), during (pressure), and after (post) pressure stress is shown. **(A)** The results of the control experiments without pressure lid (open MEA), 0 mmHg, and 10 mmHg, are presented. **(B)** The results of the test groups with 30, 60, 90 mmHg, and 90 mmHg + taurine (1 mM) are presented. **(C)** Summary of the normalized number of spikes in bursts during pressure is shown. The number of spikes in bursts before pressure (pre) was set to 1. Data are presented as box and whisker (min to max) plots. Outliers calculated by ROUT (Q = 0.1%) are shown as red circles. Mean values of the cleaned data are shown as + . Significant values are indicated by asterisks. Not significant values are labeled with ns in panels **(A,B)** and not labeled in panel **(C)**. One-way ANOVA with *post hoc* Tukey’s test was performed with GraphPad Prism Software version 7. **(A)** *F*(2, 1946) = 7.173, *p* = 0.0008; *F*(2, 847) = 0.1714, *p* = 0.8425; *F*(2, 939) = 5.471, *p* = 0.0043; **(B)** *F*(2, 1370) = 18.86, *p* < 0.0001; *F*(2, 1495) = 6.209, *p* = 0.0021; *F*(2, 1432) = 34.8, *p* < 0.0001; *F*(2, 1551) = 8.43, *p* = 0.0002; **(C)** *F*(4, 2885) = 57.32, *p* < 0.0001. *n*_retinae_ = 5/group, *n*_cells_ = 152–430/group, *n*_recordings_ = 16/experiment.

During experiments with highly increased pressure of 60 and 90 mmHg, salves of spikes (bursts) fired by the RGCs were observed. A burst analysis was performed and the RGC bursting behavior on single cell level after spike sorting was analyzed and plotted for each experimental phase (Figure 4). The percentage of spikes in bursts in relation to all the spikes fired by an RGC are shown. The control without pressure lid (open MEA) showed a slight increase of spikes fired in bursts (pre: 17.38 ± 22.83%, pressure: 22.37 ± 22.53%, post: 21.14 ± 20.63). In the 0 mmHg control group, the incidence of bursts did not significantly change (pre: 15.91 ± 22.00%, pressure: 16.92 ± 20.49%, post: 16.88 ± 19.25%). In the 10 mmHg control group, the incidence of bursts slightly increased over time (pre: 12.11 ± 19.33%, pressure: 17.14 ± 20.57%, post: 17.77 ± 20.05%) (Figure 4A). Under a pressure level of 30 mmHg, the incidence of bursts increased by 55% from 12.46 ± 18.75% to 19.37 ± 18.52% (Figure 4B) and persisted after lowering the pressure (20.53 ± 19.59%). Under 60 mmHg, the incidence of bursts increased by 39% from 11.02 ± 16.19% to 15.35 ± 18.13%; after pressure, the mean incidence of bursts was 13.98 ± 16.53%. Under 90 mmHg, the incidence of bursts increased by 108% from 9.90 ± 15.58% to 20.60 ± 20.42% and remained at a high level during the post-phase (21.21 ± 20.18%). Bursting behavior of the RGCs under pressure in the presence of taurine were different to the results under 90 mmHg without taurine: instead of an increase under pressure stress, the incidence of bursts decreased by 16% during, and by 20% after application of 90 mmHg (pre: 17.81 ± 23.97%, pressure: 15.03 ± 21.90%, post: 12.09 ± 18.93%). The addition of taurine significantly counteracted the effect of high pressure on the RGC bursting behavior.

Figure 4C shows a summary of the normalized spikes-in-bursts rate during pressure stress for each pressure level with the initial number before pressure stress being set to 1. The number of spikes in bursts was 140% under 10 mmHg, 155% under 30 mmHg, 139% under 60 mmHg, 208% under 90 mmHg, and 84% under 90 mmHg in addition of taurine (Figure 4C). Comparison of the number of spikes in bursts between the different pressure levels revealed a significantly higher incidence of bursts under 90 mmHg: more bursts occurred and the number of bursts stayed at a high level even after the pressure was reduced back to control conditions.

**FIGURE 5:**
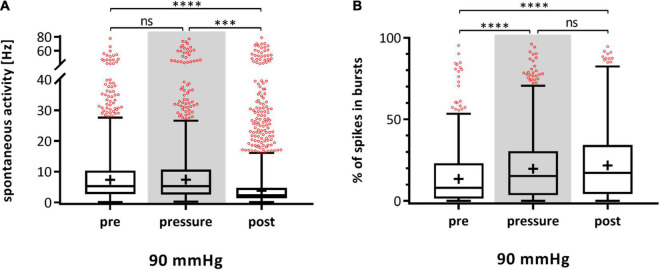
Spontaneous activity and bursting behavior of RGCs in dark adapted retinae under 90 mmHg. The spontaneous firing frequency [Hz] **(A)** and the number of RGC spikes in bursts [%] **(B)** before (pre), during (pressure), and after (post) pressure stress of 90 mmHg is shown. The MEA experiments were carried out with dark adapted retinae under dim red light. Data are presented as box and whisker (min to max) plots. Outliers calculated by ROUT (Q = 0.1%) are shown as red circles. Mean values of the cleaned data are shown as + . Significant values are indicated by asterisks. Not significant values are labeled with ns. One-way ANOVA with *post hoc* Tukey’s test was performed with GraphPad Prism Software version 7. **(A)** *F*(2, 1922) = 12.79, *p* < 0.0001; **(B)** *F*(2, 1922) = 14.5, *p* < 0.0001. *n*_retinae_ = 5, *n*_cells_ = 262, n_recordings_ = 90 **(A)**, 70 **(B)**.

Multielectrode array experiments with dark adapted retinae were performed under dim red light and the effect of high pressure stress (90 mmHg) on RGC activity was analyzed. The spontaneous firing frequency did not change significantly from pre- (11.64 ± 12.77 Hz) to pressure-phase (11.10 ± 13.06 Hz), but decreased after pressure stress by 25% (8.38 ± 12.62 Hz) (Figure 5A). The incidence of bursts also increased under pressure by 37% and stayed at a high level throughout the post-phase (pre: 16.38 ± 19.22%, pressure: 22.38 ± 21.99%, post: 22.67 ± 21.26%) (Figure 5B).

**FIGURE 6:**
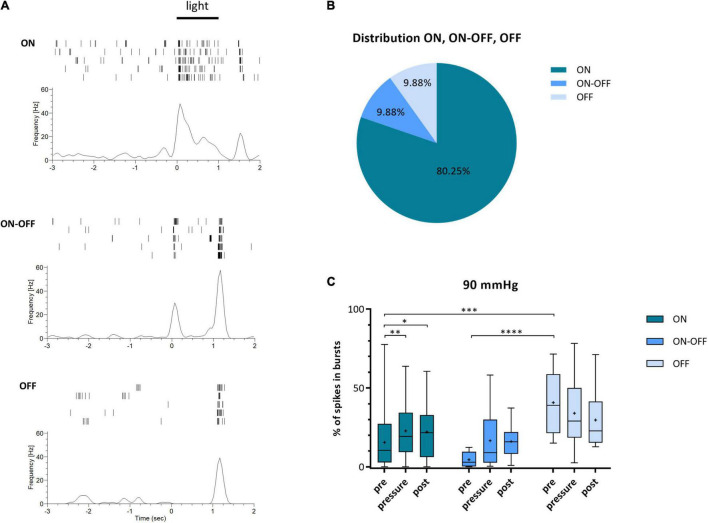
Classification of RGCs into ON, ON-OFF, and OFF cells and their type specific bursting behavior under 90 mmHg. RGCs were categorized according to their response to light stimulation into the three categories ON, ON-OFF, and OFF. **(A)** Characteristic examples of ON-, ON-OFF-, and OFF responses of RGCs after light stimulation (full-field, 1 s, 160 lm) are shown. **(B)** The distribution of the three RGC categories ON (dark blue), ON-OFF (blue), and OFF (light blue) is presented. Uncategorized cells are not included. **(C)** The number of spikes in bursts [%] before (pre), during (pressure), and after (post) pressure stress of 90 mmHg for ON (dark blue), ON-OFF (blue), and OFF (light blue) RGCs is shown. Data are presented as box and whisker (min to max) plot. Mean values are shown as + . Significant values are indicated by asterisks. Two-way ANOVA with *post hoc* Tukey’s test was performed with GraphPad Prism Software version 7. **(C)** *F*(4, 156) = 2.12, *p* = 0.0809. *n*_retinae_ = 5, *n*_cells_ = 83.

According to their light response, the recorded RGCs were roughly sorted into three categories: ON, ON-OFF, and OFF. Characteristic examples of spike trains during light stimulation for the three categories are shown in Figure 6A. However, the different RGC types were not distributed equally. Excluding the uncategorized responses (18%), the vast majority of RGC light responses were categorized as ON with 80.25% (Figure 6B). ON-OFF and OFF responses were identified in equal parts (9.88% ON-OFF, 9.88% OFF). The ON cells revealed a significant increase in bursts of 47% during increased pressure and the incidence stayed at a high level after pressure stress (pre: 15.48 ± 15.04%, pressure: 22.78 ± 17.47%, post: 22.10 ± 16.60%) (Figure 6C). ON-OFF cells also showed an increase in bursts during and after pressure stress (pre: 4.53 ± 4.92%, pressure: 16.55 ± 20.18%, post: 16.08 ± 11.00%). Overall, the number of spikes in bursts increased by 265% under pressure. OFF cells, however, did not show an increase in bursts during and after pressure stress: under 90 mmHg, the number of spikes in bursts slightly decreased by 17% from 40.75 ± 20.58% (pre) to 33.96 ± 23.73% (pressure) and further to 29.66 ± 19.76% (post).

**FIGURE 7:**
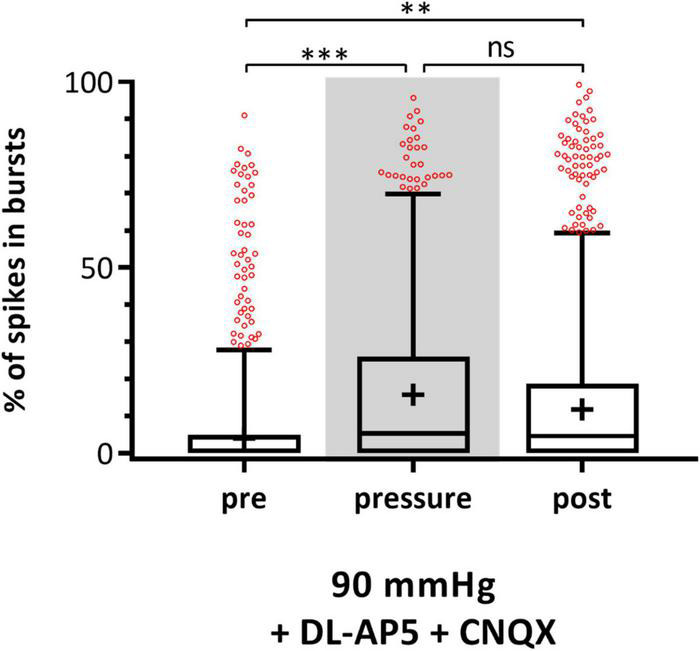
Effect of glutamate receptor blockers on RGC bursting behavior under 90 mmHg. The number of spikes in bursts [%] before (pre), during (pressure), and after (post) pressure stress of 90 mmHg in addition of the glutamate receptor blockers DL-AP5 and CNQX is shown. Data are presented as box and whisker (min to max) plots. Outliers calculated by ROUT (*Q* = 0.1%) are shown as red circles. Mean values of the cleaned data are shown as + . Significant values are indicated by asterisks. Not significant values are labeled with ns. One-way ANOVA with *post hoc* Tukey’s test was performed with GraphPad Prism Software version 7. *F*(2, 1365) = 6.814, *p* = 0.0011. *n*_retinae_ = 3, *n*_cells_ = 229, *n*_recordings_ = 48.

To investigate the origin of bursts during increased pressure, MEA experiments with 90 mmHg in addition of the glutamate receptor blockers DL-AP5 and CNQX were performed. The blockage of the glutamatergic input resulted in lesser fluctuations in the local field potentials (LFPs) and lead to a flat baseline (data not shown). The incidence of bursts during pre-, pressure-, and post-phase is presented in Figure 7. Under 90 mmHg, the number of spikes in bursts significantly increased by 51% from 13.24 ± 21.59% (pre) to 19.94 ± 24.95% (pressure) and stayed at a high level after pressure (post: 18.43 ± 24.93%).

### Live/Dead Staining

**FIGURE 8:**
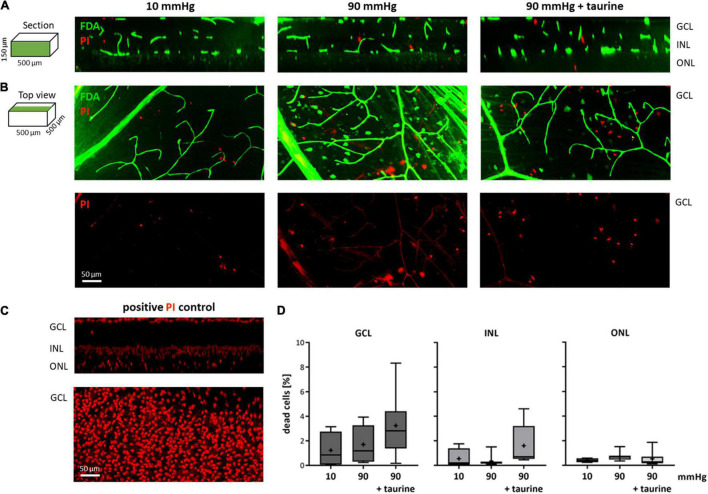
Live/dead stained retinae after pressure stress analyzed *via* TPLSM. Retinae were live/dead double stained with FDA (green) and PI (red) and analyzed using a two photon microscope. Retinae that endured 10 mmHg (control), 90 mmHg, and 90 mmHg + taurine (1 mM) were analyzed. **(A)** Representative examples of sections through image stacks showing GCL, INL, and ONL are presented. **(B)** Representative examples of the top view of image stacks onto the GCL. **(C)** Representative section and top view of a retina after 4 h hypoxic stress (positive PI control). **(D)** Number of dead cells related to the number of dead cells in the positive PI control [%]. Data are presented as box and whisker (min to max) plots for GCL (dark gray), INL (gray), and ONL (light gray). Mean values are shown as + . No significant values were identified. One-way ANOVA with *post hoc* Tukey’s test was performed with GraphPad Prism Software version 7. **(D)** *F*(2, 17) = 1.863, *p* = 0.1855; *F*(2, 17) = 2.593, *p* = 0.1040; *F*(2, 17) = 0.6987, *p* = 0.5110. *n*_retinae_ = 4/group, *n*_stacks_ = 2/retina.

Retinae were live/dead double stained to investigate the survival rate of the cells under pressure stress. The PI staining was successful throughout the tissue. FDA, however, mainly stained the blood vessels, nerve fiber layer (NFL), and only single cells (Figures 8A,B). Therefore, the number of dead cells could not be normalized to the number of living cells; instead, it was related to the mean number of dead cells of PI positive controls (Figure 8C). Here, a total number of 1,775.75 ± 180.00 cells for GCL, 4,925.00 ± 491.00 for INL, and 1,716.25 ± 1,003.00 for ONL was determined. Compared to literature values (Jeon et al., 1998), PI staining in the positive controls achieved an efficiency of 87%.

In the GCL, the number of dead cells changed from 1.23 ± 1.46% in the control group to 1.70 ± 1.52% under 90 mmHg and 3.24 ± 2.49% under 90 mmHg + taurine (Figure 8D). In the INL, the number of dead cells was 0.55 ± 0.81% under 10 mmHg (control), 0.36 ± 0.47% under 90 mmHg, and 1.61 ± 1.65% under 90 mmHg + taurine. In the ONL, the number of dead cells changed from 0.41 ± 0.17% to 0.71 ± 0.36% under 90 mmHg and 0.52 ± 0.59% under 90 mmHg + taurine.

## Discussion

The retinal *ex vivo* pressure model established here serves as a glaucoma acute model for short-time increased IOP. The electrical RGC activity could be continuously measured before, during, and after pressure stress. Furthermore, the effect of taurine (1 mM) on the firing behavior was investigated. The experimental setup enabled a variation of hydrostatic pressure inside the chamber without influencing oxygen concentration or pH of the medium.

In all three experimental phases (pre, pressure, and post) the spontaneous activity as well as the response to given stimuli persisted. There was no pressure dependent effect on the firing frequency, nor on the response rate. There were changes over time; however, these changes occurred in all groups, so that they were rather time dependent than pressure related. The retinal functionality remained even under a highly increased pressure of 90 mmHg applied for 2 h. The unsorted MEA data (channel level) revealed an increase in RGC activity under pressure stress, whereas the sorted data (cell level) showed no increase in the RGCs’ firing frequency. The increase in recorded activity at channel level was most likely a physical effect. Due to the increased hydrostatic pressure, the retina was pressed against the MEA electrodes so that the contact between tissue and electrodes improved and more cells could be recorded. This assumption was also supported by a slight increase in the spike amplitudes under pressure that subsided after lowering the pressure during the post-phase (data not shown). In general, RGC firing behavior was very diverse causing a high variability of the MEA recording results in all the groups including the controls. Therefore, many outliers have been identified and the gain of significant results was made more difficult. For example, spontaneous firing frequency decreased significantly in the 30 mmHg and the 60 mmHg group, but not significantly in the 90 mmHg group. However, the tendency was the same in all three groups.

Other studies presented impairment of retinal functionality in the form of changes in a- and b-waves in electroretinogram (ERG) recordings: pressure elevation was achieved by cannulation of rat eyes (Bui et al., 2005) or the microbead injection method in mouse eyes (Frankfort et al., 2013). The effect of increased pressure by microbead occlusion in mice was also analyzed using MEA recordings: after 3–7 weeks of mild increased IOP (15–24 mmHg), a slight light sensitivity loss was found (Pang et al., 2015) and after 2 weeks (+ 3 mmHg), decreased spontaneous RGC activity, altered interspike interval variance, and impaired contrast sensitivity were detected (Tao et al., 2019). Della Santina et al. (2013) showed that functional changes in RGCs preceded alterations in structure after IOP elevation (15 or 30 days) in microbead-injected mouse eyes. Ou et al. (2016) used MEA recordings to investigate αRGCs after transient laser-induced ocular hypertension (14 days) in CD-1 mice and found a decline in spontaneous activity in αOFF-transient RGCs. Although retinal ischemia is unlikely after photocoagulation, an effect on the ocular perfusion pressure cannot be ruled out completely. Furthermore, αRGCs only account for about four percent of murine RGCs (Sanes and Masland, 2015). These results do not contradict the findings of our study, but encourage further investigation of the susceptibility of different RGC types to pressure stress. Sabharwal et al. (2017) discovered a reduction of the receptive field after 6–7 weeks by whole-cell voltage clamp recordings. However, except for Bui et al. (2005) these studies investigated mild chronic IOP elevation rather than severe acute pressure increase. Furthermore, the *in vivo* pressure application made it difficult to differentiate between the effect of mechanical and hypoxic stress related to impaired blood flow. In our study, the investigation of RGC functionality included the analysis of responses to full-field light stimuli. In future studies, the external light source within the experimental setup can be adjusted or replaced and a multitude of different light stimuli can be tested in order to investigate, e.g., contrast sensitivity and receptive field properties that have been proven to be affected by pressure stress (Pang et al., 2015; Sabharwal et al., 2017; Tao et al., 2019).

Although the RGC functionality remained stable during pressure stress, the bursting behavior of the cells was strikingly different. The burst analysis revealed a significantly higher incidence of bursts under a highly increased pressure of 90 mmHg. Furthermore, the change in the RGCs’ bursting behavior was not reversible. The number of spikes in bursts remained high after pressure stress. However, increased pressure did not seem to affect all RGC types equally. The MEA experiments with light stimulation revealed that the increase in bursts under pressure stress is carried by ON-, and ON-OFF RGCs, but not by OFF RGCs, suggesting that OFF RGCs are more resistant to pressure stress. Also the application of glutamate receptor antagonists DL-AP5 and CNQX resulted in an increase of bursts under pressure suggesting that the bursts were generated in the RGCs themselves and were independent of the glutamatergic input *via* AMPA/kainate and NMDA receptors.

Altered bursting behavior can also be seen in animal models of retinal degeneration. In *rd1* and *rd10* mice, bursts occur alongside of oscillatory LFPs affecting the retinal response properties after electrical stimulation (Jensen and Rizzo, 2008; Park et al., 2015). In healthy retinae, RGCs fire bursts in response to changes in the internal circuit or external visual stimuli. Bursts can improve the transmission of visual signals to the lateral geniculate nucleus and induce its synapse plasticity (Moore et al., 2011; Alitto et al., 2019). However, it still needs to be examined how the altered bursting behavior during and after high pressure stress affects the retinal functionality in the long term.

There was no significant change in the survival rate under pressure stress in none of the three investigated retinal layers. Overall, the highly increased pressure of 90 mmHg did not affect the retinal cells’ survival, nor were any neuroprotective effects observed by the addition of 1 mM taurine, which was consistent with the outcome of the MEA recordings.

Other studies also proofed the resistance of retinal cells to sheer pressure stress. Osborne et al. (2015) showed that neither constant, nor fluctuating increased hydrostatic pressure (10–100 mmHg) caused significant changes in the survival rate of human RGCs in culture. The work of Aihara et al. (2014) showed that pressure increase (+ 15/30/90 mmHg) alone did not induce cell death in primary cultures of rat RGCs; however, the susceptibility to glutamate toxicity under pressure stress was increased. Consistent results were shown by histological examinations of rat retinae (Ishikawa et al., 2010): hydrostatic pressure of 50 mmHg and 75 mmHg induced axonal swelling in the NFL. The swelling was prevented by the addition of glutamate receptor antagonists. Therefore, the authors suggested that neural degeneration under pressure stress is caused by an impaired glial glutamate metabolism.

For there was no significant impairment of the RGC survival rate under pressure stress, the investigation of the neuromodulating effect of taurine, rather than the neuroprotective effect, was of interest. The addition of taurine had no effect on the spontaneous RGC firing frequency, nor on the RGC response rate. However, the incidence of bursts during and after pressure stress was significantly altered by taurine: it caused a decrease of spikes in bursts during and after high pressure stress and therefore counteracted the effect of high pressure on the RGC bursting behavior.

A neuroprotective effect of taurine on RGCs has been shown in two different glaucomatous animal models (Froger et al., 2012, 2013). The survival rate of RGCs in DBA/2J mice and Long-Evans rats with episcleral vein occlusion was significantly increased under taurine supplementation. For the primary cells *in vitro*, taurine was added to the cell culture medium, and for the *in vivo* experiments, taurine was provided *via* the drinking water. In addition to these neuroprotective effects, the neuromodulating effects on RGCs shown here make taurine an interesting candidate for glaucoma treatment in the future.

Since hydrostatic pressure had only mild effects on the electrical activity of the retina and no effect on its survival rate, mechanosensitive receptors within the retinal tissue do not seem to play a role in reactions to acute pressure stress. However, using an *in vivo* rodent hypertension model, the transient receptor potential vanilloid 1 (TRPV1) channel was proven to be involved in RGC apoptosis induced by long pressure stress (+70 mmHg for 48 h), likely through the inflow of extracellular Ca^2+^ (Sappington et al., 2009). Furthermore, the expression of five of the six transient receptor potential (TRP) channel subfamilies and two piezo channels (Piezo1, Piezo2) was detected in the ONH (Choi et al., 2015).

Other models to investigate the effect of pressure stress on the retina are diverse and range from *in vitro* cell culture models to *in vivo* animal models with naturally or induced glaucomatous pathomechanisms. The effect of increased ocular pressure on retinal cells and explants in culture has been simulated with pressure chambers (Aihara et al., 2014; Osborne et al., 2015; Ishikawa et al., 2016) or hydrostatic pressure of liquid columns (Ishikawa et al., 2010). Elevated IOP *in vivo* has been induced *via* cannulation (Bui et al., 2005; Wang et al., 2021), microbead injection into the anterior chamber occluding the outflow of aqueous humor (Sappington et al., 2010; Frankfort et al., 2013; Pang et al., 2015; Park et al., 2019), hyaluronic acid (Moreno et al., 2005) or hypertonic saline injection (Kipfer-Kauer et al., 2010), and vein cauterization/photocoagulation (Garcia-Valenzuela et al., 1995; Ji et al., 2005; Pang et al., 2015). The optic nerve crush is another common glaucoma model, but it leads to ONH atrophy independent of the IOP level (Levkovitch-Verbin et al., 2000; Choi et al., 2015; Cameron et al., 2020). Apart from the cannulation method, by which the IOP can be adjusted reliably by the height of the liquid column, all other *in vivo* models fail to induce defined pressure levels and cause high rejection rates of animals when target pressure levels are not met. Moreover, measured effects can never be definitely attributed to the pressure stress alone. Although the influence of elevated IOP on blood flow is often avoided by checking retinal blanching or measuring the blood pressure, an impairment cannot be ruled out completely. These problems are also present in the DBA/2J mouse model (Anderson et al., 2006; Inman et al., 2006; Williams et al., 2013). The limitations of the *in vivo* models seem to be overcome in the *in vitro* models mentioned above; however, most of the studies have focused on histological and molecular investigations of the retina after pressure application rather than on the functionality.

The MEA-based pressure model presented here enables the analysis of the retinal functionality not only after, but also before and during pressure application. Experimental conditions can be precisely adjusted and the functional electrical activity of the retina can be recorded in real time. Furthermore, the effect of neuroprotective agents can easily be examined. However, the artificial conditions during MEA recordings limit the duration of an experiment to several hours. Therefore, the setup serves more as an acute model imitating conditions of glaucomatous attack rather than chronical IOP elevation.

Although high IOP is considered the main risk factor in glaucoma, we showed that the sheer mechanical stress of pressure does not seem to affect the retinal functionality in the acute model. In contrast, we showed that hypoxic stress has a dramatic effect on the RGCs’ electrical activity and their survival rate (Ingensiep et al., 2021). Hypoxia can be induced *in vivo* by impaired blood flow under high IOP (Andreeva et al., 2014; Wiemann et al., 2021). The findings of our work help to better evaluate and understand the role of pressure stress in glaucoma next to other stressors, such as hypoxia.

## Data Availability Statement

The raw data supporting the conclusions of this article will be made available by the authors, without undue reservation.

## Ethics Statement

The animal study was reviewed and approved by Institute of Laboratory Animal Science (Faculty of Medicine, RWTH Aachen University).

## Author Contributions

CI, KS, PW, and SJ contributed to conception and design of the study. CI performed the experiments, performed the data analysis, and wrote the manuscript. KS, PW, and SJ revised and edited the manuscript. SJ was responsible for funding acquisition. All authors contributed to the article and approved the submitted version.

## Conflict of Interest

The authors declare that the research was conducted in the absence of any commercial or financial relationships that could be construed as a potential conflict of interest.

## Publisher’s Note

All claims expressed in this article are solely those of the authors and do not necessarily represent those of their affiliated organizations, or those of the publisher, the editors and the reviewers. Any product that may be evaluated in this article, or claim that may be made by its manufacturer, is not guaranteed or endorsed by the publisher.
